# Long noncoding RNA matrilineal expression gene 3 inhibits hepatocellular carcinoma progression by targeting microRNA-5195-3p and regulating the expression of forkhead box O1

**DOI:** 10.1080/21655979.2021.2005986

**Published:** 2021-12-13

**Authors:** Minan Li, Hong Liao, Jian Wu, Bin Chen, Runhua Pang, Junhai Huang, Yaqing Zhu

**Affiliations:** The Third Department of Surgery, The First Affiliated Hospital of Guangzhou University of Chinese Medicine, Guangzhou, Guangdong, China

**Keywords:** MEG3, HCC, miR-5195-3p, FOXO1

## Abstract

We investigated the effect of the long noncoding RNA (lncRNA) maternally expressed gene 3 (MEG3) on hepatocellular carcinoma (HCC) tumorigenesis and progression by targeting miR-5195-3p and transcription factor forkhead box O1 (FOXO1) to identify a novel target for HCC treatment. HCC clinical samples were collected, and cell counting kit-8 (CCK-8), and transwell migration and invasion assays were performed. Furthermore, interaction was detected via double luciferase reporter and RNA pull-down assays. MEG3, miR-5195-3p, and FOXO1 expression was determined by quantitative real-time polymerase chain reaction (RT-qPCR) and Western blotting. Xenograft tumor models were established to investigate the effect of MEG3 *in vivo*. Compared with normal tissues, MEG3 expression was significantly downregulated in HCC tissues. MEG3 overexpression inhibited the viability and migration of HCC cells. Double luciferase reporter and RNA pull-down assays confirmed the binding between MEG3 and miR-5195-3p as well as between miR-5195-3p and FOXO1. RT-qPCR and Western blotting results showed that MEG3 inhibited the expression of miR-5195-3p and promoted that of FOXO1. Additionally, MEG3 overexpression inhibited HCC tumorigenesis and progression in xenograft tumor models while depletion of MEG3 exerted the opposite way. Therefore, the lncRNA MEG3 inhibits HCC tumorigenesis and progression through the miR-5195-3p/FOXO1 signaling axis.

## Introduction

Hepatocellular carcinoma (HCC) affects thousands of patients worldwide every year [1]. HCC is highly malignant among common tumors, and its mortality rate ranks third in the world. It has a number of molecular characteristics, including distant metastasis and local invasiveness, especially intrahepatic metastasis, which is very common after surgery [2]. Although HCC therapy has made considerable progress and development in terms of surgical techniques, chemotherapy drugs and targeted drugs in recent years, HCC still has a very high incidence and mortality rate, posing a serious threat to human health [3]. Therefore, the molecular mechanism of HCC, prognostic diagnostic tumor indicators, and drug targets for targeted therapy require further in-depth study [4].

In recent years, long non-coding RNA (lncRNA) has attracted increasing attention and research from many aspects in the biomedical field [5]. In the study of many diseases, some lncRNAs that function as diagnostic and prognostic tools have gradually emerged. Recent findings have also confirmed that lncRNAs have a variety of biological functions, including regulating chromosome recombination, controlling gene transcription, and mRNA processing [6]. LncRNAs involved in HCC also have a wide range of mechanisms, including DNA inactivation, methylation, transcriptional promotion, and activation of other RNA molecules [7]. Moreover, studies have shown that lncRNA can undergo various epigenetic modifications, including methylation and ubiquitination, and act together with microRNAs (miRNAs) to form a network regulatory structure [8].

Maternally expressed gene 3 (MEG3), located on human chromosome 14q32.3, is involved in inhibiting the growth of various malignant tumor cells and is closely related to the occurrence, development, metastasis and other biological processes of tumors [9]. Changing expression level of lncRNA MEG3 can affect the metabolic process of body cells [10]. MEG3 can also reduce the abundance of murine double minute 2 (MDM2), a negative regulator of p53, thus affecting the expression of tumor suppressor p53 [11]. It can selectively activate the downstream target gene of p53, growth differentiation factor-15 (GDF15), and participate in the occurrence and development of malignant tumors including HCC [11,12]. This study aims to further improve the mechanism of MEG3 in HCC on the basis of previous studies.

In this study, we aimed to find the function and mechanism of MEG3 in HCC. Cell viablity, migration, and invasion were combined to evaluate function of MEG3 and target gene *in vitro*, while tumor growth was for *in vivo* experiment. These findings may provide novel targets for the treatment of HCC.

## Materials and methods

### HCC samples

In total, 40 pairs of HCC and adjacent normal tissues were surgically removed from patients undergoing primary HCC surgery in The First Affiliated Hospital of Guangzhou University of Chinese Medicine. Written informed consent was obtained from all patients, and the study was approved by the ethics committee of The First Affiliated Hospital of Guangzhou University of Chinese and was conducted in accordance with the Declaration of Helsinki.

### Cell culture

Human normal liver cell line QSG-7701 and human HCC cell lines HepG2, Hep3B, Huh7, and MHCC-97 H were purchased from the American Type Culture Collection (Manassas, VA, USA). The different cell lines were cultured in Dulbecco’s modified Eagle’s medium (Solarbio, China) supplemented with 10% fetal bovine serum (Hyclone, USA) in a 5% CO_2_ incubator at 37°C [13].

### Cell transfection

All the plasmids (vector, MEG3, mimic-NC, miR-5195-3p mimic, si-nc, si-MEG3, sh-nc, and sh-forkhead box O1 (FOXO1)) were purchased from Sangon Biotech. Co., Ltd. (Shanghai, China). The cells were transfected using lipo3000 (Biosharp, China) according to the manufacturer’s instructions [13].

### Cell counting kit 8 (CCK-8) assay

The transfected cells were diluted in 96-well plates at a density of 5 × 10^3^ cells per well. CCK-8 regeant (10 μL; Solarbio) was added to each well and incubated for 1 h at 37°C in the dark [14]. The absorbance was recorded at 450 nm with a microplate reader. After the detection values were obtained, the cell viability and proliferation rates were calculated.

### Transwell assay

The transfected cells were diluted in 24-well plates at 2 × 10^5^ cells per well. Cell suspension (150 μL) was added to the Transwell upper chamber (Millipore, USA). 500 μL complete mediun was added to the Transwell lower chamber [15]. 24 h later, the cells were fixed in methanol for 15 min and stained with crystal violet for 30 min. Cell migration and invasion was observed under a microscope (Leica, Germany).

### RNA extraction and real-time polymerase chain reaction (PCR) analysis

Total cell RNA was extracted using RNA Fast kit (Solarbio), and cDNA synthesis was performed using HiScript®II Reverse Kit (Vazyme, China). Quantitative real-time PCR (RT-qPCR) was performed using SYBR Green PCR Mix (Biosharp) on an IQ5 PCR System (Bio-Rad Laboratories, Hercules, CA, USA). The program used was as follows: 94°C for 1 min, 94°C for 20 s, 58°C for 30 s, 72°C for 30 s, with 40 cycles. The primers used were as follows: MEG3, sense: 5ʹ-GTCAATACGGATCATATCCT-3ʹ, antisense: 5ʹ-ACTGCAACTGACCATTCTAC-3ʹ; miR-5195-3p, sense: 5ʹ-AGCAGTTTATTGACATAAATCAA-3ʹ, antisense: 5ʹ-CGTGAACTCGGTCTATGATTCA-3ʹ; FOXO1, sense: 5ʹ-AACTTTCGCTTAGTGGAACGT-3ʹ, antisense: 5ʹ-ACCCTCATACCTTTGGAACAG-3ʹ; U6, sense: 5ʹ-CTCGCTTCGGCAGCACA-3ʹ, antisense: 5ʹ-ACGCTTCACGAATTTGC-3ʹ; and GAPDH, sense: 5ʹ-AGAAGGCTGGGGCTCATTTG-3ʹ, antisense: 5ʹ-AGGGGCCATCCACAGTCTTC-3ʹ. The relative expression of the target genes was calculated using 2^−ΔΔCt^ [16]. U6 and GAPDH were used as the internal control for miRNA and mRNA, respectively.

### RNA pull-down assay

Biotinylated miR-5195-3p (biotin-miR-5195-3p) and nc (biotin-nc) were transfected into HCC cells. After 48 h, the transfected cells were treated with lysis buffer for 10 min and harvested. The cells were then incubated with M-280 Streptavidin magnetic beads (Invitrogen, Waltham, MA, USA) at 4°C for 3 h. Precooled lysis buffer was used to wash the cells, and RT-qPCR was performed to measure the enrichment of MEG3 and FOXO1.

### Western blotting

The transfected cells were collected and resuspended in 0.5 mL radioimmunoprecipitation assay buffer (Solarbio,). Cell lysates were placed on ice for 30 min, followed by centrifugation at 10,000 g for 20 min. Protein concentration was determined using the bicinchoninic acid kit (Thermo Fisher Scientific, USA). Protein samples (20 μg) were electrophoresed via sodium dodecyl sulfate-polyacrylamide gel electrophoresis and transferred onto a polyvinylidene Fluoride membrane. The The membrane was blocked with 5% nonfat milk in Tris-buffered saline with Tween 20 at room temperature for 2 h and then probed with FOXO1 and GAPDH primary antibody (1:2000 dilution; Boster, China) at 4°C overnight. The secondary antibody used for detection was a horseradish peroxidase-conjugated anti-rabbit IgG, and the protein expression was detected using an ECL chemiluminescence detection kit (Boster).

### Double luciferase reporter assay

Wild-type (wt) MEG3 or mutant (mut) sequence of FOXO1 were synthetized and coloned into a GV272 reporter vector. The wt or mutant MEG3 or FOXO1 vectors were transfected into HepG2 and Huh7 cells which have been transfected with miR-5195-3p mimic or control vectors. After transfection for 24 h, cells were collected and analyzed using a dual-luciferase reporting system.

### Xenograft tumor model

Female nude mice (four to five weeks old) were purchased from Beijing Vital River Laboratory Animal Technology Co., Ltd. and randomly divided into si-nc, si-MEG3, vector and MEG3 overexpression groups (n = 3 per group). Stably transfected cells were subcutaneously injected into the mice (1 × 10^6^ cells per mouse), and tumor size was measured weekly [17]. After four weeks, the mice were anesthetized via intraperitoneal injection of 1% pentobarbital sodium (160 mg/kg) and sacrificed via asphyxiation. Liver tumor tissues were isolated and photographed, and their volume and weight were measured. Tumor volume was calculated as follows: V (mm^3^) = (length×width^2^)/2.

### Statistical analysis

GraphPad Prism 5.0 was used for statistical analysis. The data were expressed as the mean ± standard deviation.The *t*-test was used for comparison between the two groups and one-way analysis of variance was used among multiple groups. Statistical significance was set at *P* < 0.05.

## Results

### lncRNA MEG3 is downregulated in tumor tissues and cell lines of HCC

**Figure 1.**
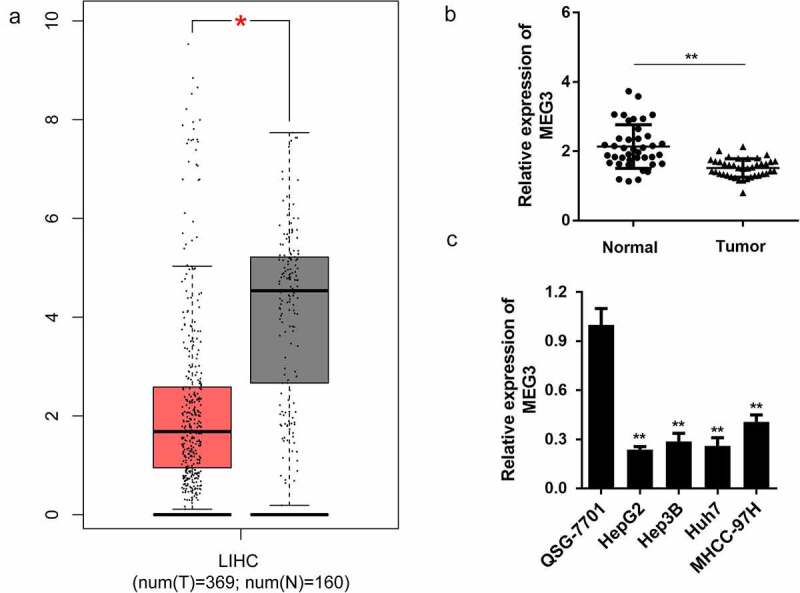
LncRNA MEG3 is downregulated in HCC tissues and cell lines

First, bioinformatics analysis indicated that MEG3 lncRNA expression in HCC samples was significantly downregulated compared to that in normal tissues (Figure 1(a)). To further confirm this, we detected MEG3 expression in tumor and normal tissues in patients with HCC. Interestingly, MEG3 expression was significantly downregulated in tumor tissues compared with normal tissues (Figure 1(b)). Furthermore, we determined the expression level of MEG3 in liver cell lines, and found that it was significantly lower than that of the normal liver cell line QSG-7701 (Figure 1(c)). Among the four liver cancer cell lines, HepG2 and Huh7 expressed the lowest MEG3 abundance. Therefore, HepG2 and Huh7 were used in subsequent experiments.

(a) Bioinformatics analysis of differentially expressed genes in HCC and normal tissues. (b) MEG3 expression level in liver cancer and normal tissues was detected by qPCR. (c) MEG3 expression level in HCC and normal cell lines was evaluated by qPCR. *P < 0.05, **P < 0.01.

### Dysregulated MEG3 affects tumor progression in HepG2 and Huh7 cells

**Figure 2.**
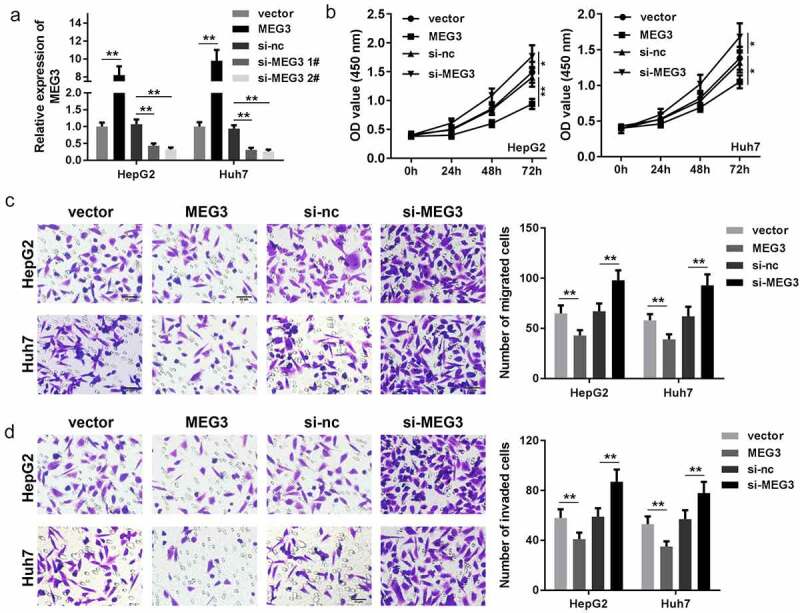
MEG3 affects the proliferation, migration, and invasion of HCC cell lines HepG2 and Huh7. (a) qPCR was performed to detect MEG3 expression in HepG2 and Huh7 cell lines. (b, c) CCK-8 was used to detect HCC cell proliferation after transfection. (d, e) Transwell assay was used to assess the migration and invasion of HCC cells. *P < 0.05, **P < 0.01

In order to study the function of MEG3 in HCC, MEG3 of HepG2 and Huh7 cells were upregulated and downregulated separately, RT-qPCR results indicated that compared with the vector group, transfection of MEG3-overexpressing vector significantly enhanced MEG3 expression while knockdown of MEG3 exerted the opposite way (Figure 2(a)). Cell viability was then determined via the CCK-8 assay. The results indicated that elevated MEG3 suppressed cell viability of both HepG2 and Huh7 cells while cell viability was promoted by suppressing MEG3 (Figure 2(b) and (c)). Transwell assay indicated that MEG3 overexpression inhibited HCC cell migration and invasion, which were accelerated by downexpression of MEG3 (Figure 2(d) and (e)). These data reveal that MEG3 inhibits HCC cell proliferation, migration, and invasion.

### MEG3 targets miR-5195-3p

**Figure 3.**
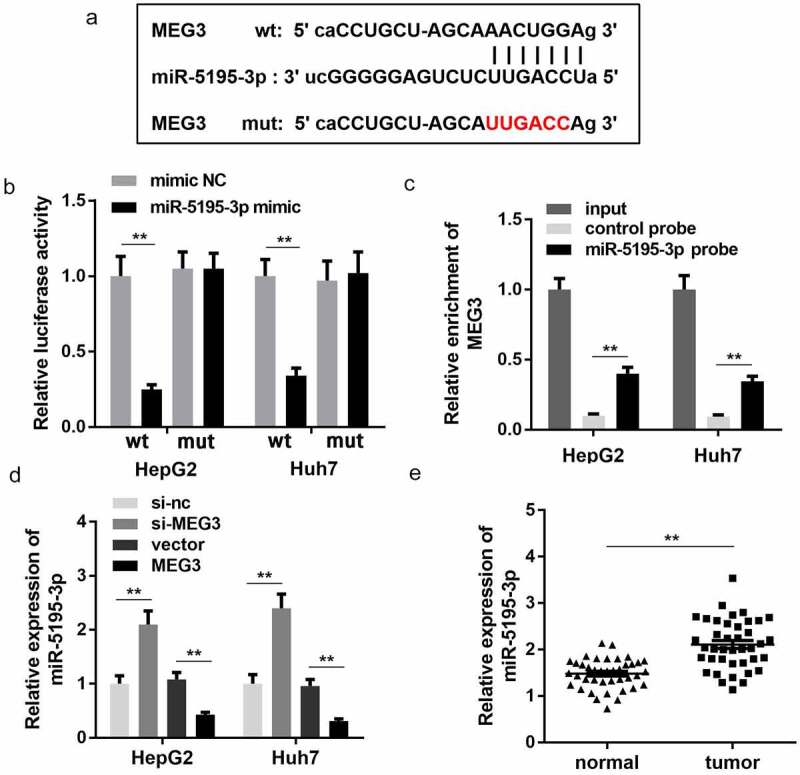
MEG3 sponges miR-5195-3p in HCC cells. (a) The binding site between MEG3 and miR-5195-3p was predicted by bioinformatics analysis. (b) Luciferase reporter assay was used to confirm the interaction between MEG3 and miR-5195-3p. (c) RNA pull-down assay was performed to determine whether MEG3 sponges miR-5195-3p. (d) MiR-5195-3p expression level was detected in MEG3 overexpression or knockdown group using qPCR. (e) qPCR was performed to evaluate the expression of miR-5195-3p in HCC tissues. **P < 0.01

In order to further study the mechanism of MEG3 in HCC progression, we first predicted the miRNA that MEG3 binds to. Bioinformatics results showed that MEG3 could potentially bind to miR-5195-3p (Figure 3(a)). Luciferase reporter assay further confirmed the binding relationship between MEG3 and miR-5195-3p. miR-5195-3p mimic transfection significantly inhibited the luciferase activity of the reporter carrying the wt MEG3 sequence while there was no change in luciferase activity of the mut group (Figure 3(b)). Meanwhile, RNA pull-down assay indicated that the biotin labeled miR-5195-3p probe could enrich the expression of MEG3, which indicates their interaction (Figure 3(c)). Moreover, the expression level of miR-5195-3p was notably increased upon MEG3 knockdown notably but was reduced upon MEG3 overexpression (Figure 3(d)). At the same time, miR-5195-3p expression was significantly upregulated in tumor tissues compared with normal tissues in HCC (Figure 3(e)).

### miR-5195-3p targets FOXO1 in HCC cells

**Figure 4.**
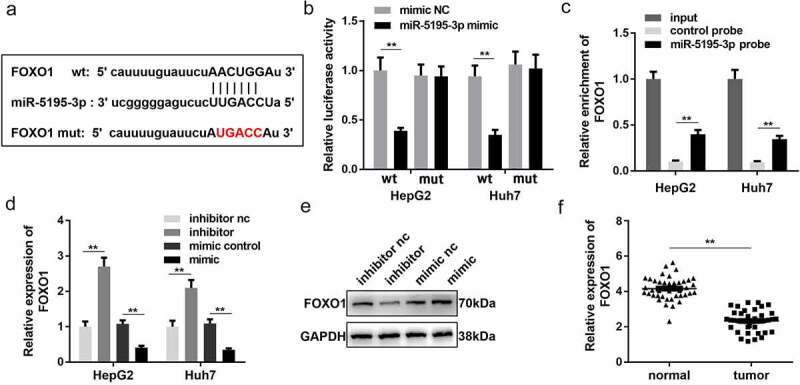
FOXO1 is a target gene of miR-5195-3p. (a) The binding site between miR-5195-3p and FOXO1 was predicted by bioinformatics analysis. (b) Luciferase reporter assay was used to confirm the interaction between FOXO1 and miR-5195-3p. (c) RNA pull-down assay was performed to determine whether miR-5195-3p targets FOXO1. (d, e) FOXO1 expression level was detected in miR-5195-3p overexpression or knockdown group using qPCR and Western blotting. (f) qPCR was performed to evaluate the expression of FOXO1 in HCC tissues. **P < 0.01

In order to further study the mechanism of MEG3 in HCC, we analyzed the downstream target genes of miR-5195-3p. Bioinformatics results showed that FOXO1 could be combined with miR-5195-3p (Figure 4(a)). Detection of luciferase reporter gene expression and RNA pull-down assay confirmed the binding between FOXO1 and miR-5195-3p (Figure 4(b) and (c)) in HepG2 and Huh7 cells. RT-qPCR and Western blotting confirmed that the effect of the miR-5195-3p mimic was consistent with that of the miR-5195-3p inhibitor and could significantly inhibit the expression of FOXO1 (Figure 4(d,e)). Moreover, compared with normal tissues, FOXO1 was significantly downregulated in HCC tissues (figure 4(f)). Based on these results, we further speculated that MEG3 might inhibit HCC development by regulating miR-5195-3p and FOXO1 expression.

### MiR-5195-3p and FOXO1 knockdown both reverse the effect of MEG3 on HCC cell proliferation, migration, and invasion

**Figure 5.**
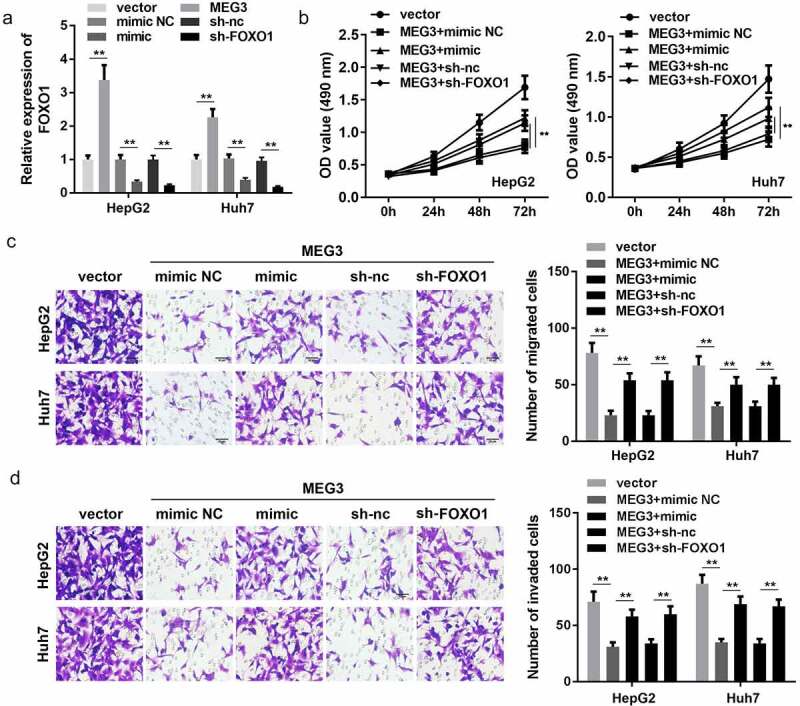
Co-expression of MEG3 and FOXO1 inhibits HCC progression in liver cancer cell lines. (a) qPCR was performed to detect FOXO1 expression in HepG2 and Huh7 cell lines. (b) CCK-8 was used to detect HCC cell proliferation after transfection. (c, d) Transwell assay was used to assess HCC cell migration and invasion. **P < 0.01

Rescue experiments were performed to further confirm the interaction of MEG3 with miR-5195-3p and FOXO1. qPCR results indicated that MEG3 overexpression promoted the expression of FOXO1, while miR-5195-3p overexpression and FOXO1 knockdown significantly inhibited FOXO1 expression (Figure 5(a)). Subsequently, functional studies, such as CCK-8 and transwell assay, were carried out and indicated that miR-5195-3p or FOXO1 knockdown reversed the effect of MEG3 on the proliferation, migration, and invasion of HCC cells (Figure 5(b-d)).

### *MEG3 affects HCC progression* in vivo

**Figure 6.**
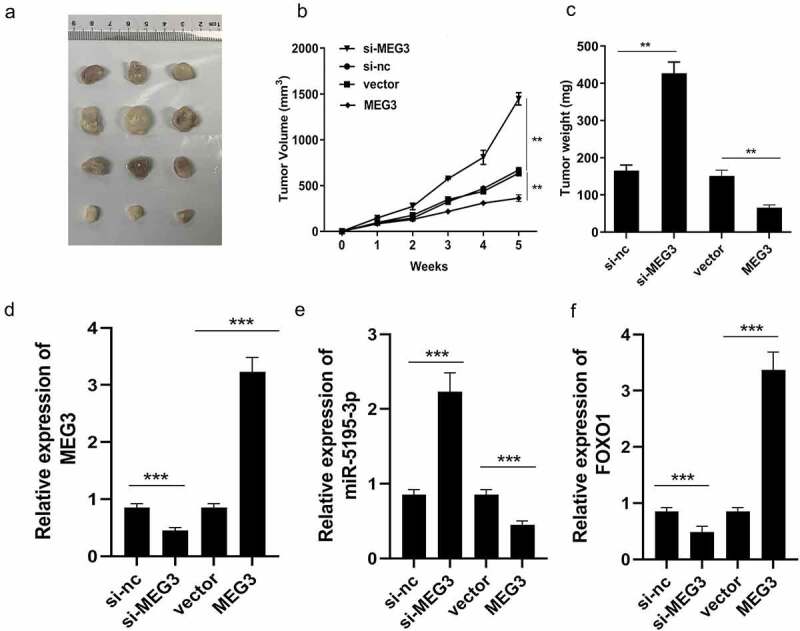
Xenograft tumor model demonstrates that overexpression of MEG3 inhibites the HCC progression. (a) The images of tumor in two groups. (b) The growth curve was established. (c) Tumors weight in each group was detected. (d-f) MEG3, miR-5195-3p and FOXO1 expression level was detected in the tumor tissues using qPCR. **P < 0.01, ***P < 0.001

To further confirm the function of MEG3, an *in vivo* experiment was performed. We successfully established the mouse allograft model, and the results showed that the tumor weight and volume of the MEG3 overexpression group decreased significantly when compared with those of the vector control group (Figure 6(a-c)). Meanwhile, in the MEG3 overexpression group, expression of miR-5195-3p was significantly inhibited, while that of MEG3 and FOXO1 was significantly enhanced, leading to an increased HCC inhibition (Figure 6(d-f)). Depletion of MEG3 exerted the opposite way on tumor weight and volume as well as MEG3 and FOXO1 expression (Figure 6(a-f)). The xenograft tumor model demonstrates that overexpression of MEG3 inhibits HCC progression and knockdown of MEG3 suppresses aggressiveness of HCC *in vivo*.

## Discussion

In this study, we found that MEG3 expression was significantly downregulated in HCC tissues, and this was closely related to tumor development, invasion, and metastasis. MEG3 overexpression suppressed aggressiveness of HCC *in vitro* and *in vivo* while inhibition of MEG3 exerted the opposite function. Furthermore, MEG3 could bind with miR-5195-3p to regulate FOXO1 expression, thereby affect cellular functions of HCC cells.

Accumulating evidence suggested that aberrant expressed lncRNAs may participate in process of HCC development [18]. Since 2009, scholars have found that MEG3 may be related to the occurrence and development of HCC [12,19]. In the past decade, MEG3 has been proved to have inhibitory effects on HCC *in vivo* and *in vitro* [12,19,20]. Our data also verified that HCC was suppressed by overexpressed MEG3, and aggravated by inhibition of MEG3 *in vitro* and *in vivo.*

In recent years, increasing studies have shown that dysregulation of miRNAs exists in a variety of solid tumors such as HCC and is closely related to the diagnosis, stage, progression, prognosis, and treatment response of tumors, suggesting that miRNAs may be involved in tumor occurrence and development [21]. In addition, with the in-depth research on the mechanism of competitive endogenous RNAs (ceRNA) [22], more and more miRNAs have been found to be able to be regulated by MEG3 to affect the process of HCC [23–25]. Therefore, it is crucial to improve the miRNA network bined with MEG3 and confirm its regulation of HCC. Multiple studies have reported that the expression of miR-5195-3p was downregulated in cervical, triple-negative breast, and colon cancer, which reduces the chemotherapy sensitivity of tumor cells to paclitaxel and inhibits the TGF-β signaling pathway and cell cycle progression, leading to malignant tumor progression [26]. However, the relationship between miR-5195-3p and HCC has not been studied and remains unclear. To elucidate the mechanism underlying MEG3 function, we determined the miRNA sponged by MEG3. Bioinformatics and experimental analysis confirmed that MEG3 can target miR-5195-3p in HCC cells. Furthermore, our data demonstrated that suppression of cell viability, migration, and invasion induced by overexpression of MEG3 were dramatically reversed by miR-5195-3p mimic vectors; demonstrating MEG3 inhibits aggressiveness of HCC by regulating miR-5195-3p.

miRNAs target and inhibit the expression of their target genes [27]. Identifying and validating the target genes of miRNAs are critical for elucidating the specific mechanisms of miRNAs in certain diseases [28]. FOXO1 was identified to be a direct target of miR-5195-3p in HCC cells. FOXO1 belongs to a conserved family of transcription factors, the FOX family [29–31]. FOXO1 transcription factors are important intracellular transcription factors whose activity is involved in cell proliferation, differentiation, stress response, and metabolism [32–34]. In tumor studies, abnormal expression of FOXO1 has been associated with tumorigenesis, tumor-induced angiogenesis and metastasis, tumor cell tolerance to stress, and tumor cell metabolic homeostasis [35,36]. Dysregulated FOXO1 has been confirmed to function as tumor suppressor in HCC [37,38]. In this study, we found that MEG3 took effect through sponging miR-5195-3p to suppress the FOXO1 expression. Our study demonstrated that MEG3 regulated FOXO1 through miR-5195-3p in HCC.

## Conclusion

In summary, the expression level of MEG3 is significantly downregulated in HCC cells and tissues, and this reduced expression is closely related to HCC progression. MEG3 inhibits cell proliferation and metastasis by regulating the expression of miR-5195-3p and FOXO1 in HCC cells, and inhibits tumor growth *in vivo*. Therefore, MEG3 may be a potential clinical drug target for HCC treatment. However, the more precise regulation mechanism in the process of HCC metastasis and recurrence needs to be further studied.

## Highlights

- lncRNA MEG3 is dowregulated in HCC tissues and cells.
- Overexpression of MEG3 suppresses tumor growth *in vitro* and *in vivo*.
- Upregulation of MEG3 contributes to inhibit aggressiveness of HCC by modulating the miR-5195-3p/FOXO1 axis
